# Synthesis of 3D Hexagram-Like Cobalt–Manganese Sulfides Nanosheets Grown on Nickel Foam: A Bifunctional Electrocatalyst for Overall Water Splitting

**DOI:** 10.1007/s40820-017-0160-6

**Published:** 2017-10-13

**Authors:** Jingwei Li, Weiming Xu, Jiaxian Luo, Dan Zhou, Dawei Zhang, Licheng Wei, Peiman Xu, Dingsheng Yuan

**Affiliations:** School of Chemistry and Materials Science, Jinan University, Guangzhou, 510632 People’s Republic of China

**Keywords:** Bifunctional electrocatalysts, Oxygen evolution reaction, Hydrogen evolution reaction, Cobalt–manganese sulfides, Water splitting

## Abstract

The exploration of low-cost and efficient bifunctional electrocatalysts for oxygen evolution reaction and hydrogen evolution reaction through tuning the chemical composition is strongly required for sustainable resources. Herein, we developed a bimetallic cobalt–manganese sulfide supported on Ni foam (CMS/Ni) via a solvothermal method. It has discovered that after combining with the pure Co_9_S_8_ and MnS, the morphologies of CMS/Ni have modulated. The obtained three-dimensionally hexagram-like CMS/Ni nanosheets have a significant increase in electrochemical active surface area and charge transport ability. More than that, the synergetic effect of Co and Mn has also presented in this composite. Benefiting from these, the CMS/Ni electrode shows great performance toward hydrogen evolution reaction and oxygen evolution reaction in basic medium, comparing favorably to that of the pure Co_9_S_8_/Ni and MnS/Ni. More importantly, this versatile CMS/Ni can catalyze the water splitting in a two-electrode system at a potential of 1.47 V, and this electrolyzer can be efficiently driven by a 1.50 V commercial dry battery.

**Electronic supplementary material:**

The online version of this article (doi:10.1007/s40820-017-0160-6) contains supplementary material, which is available to authorized users.

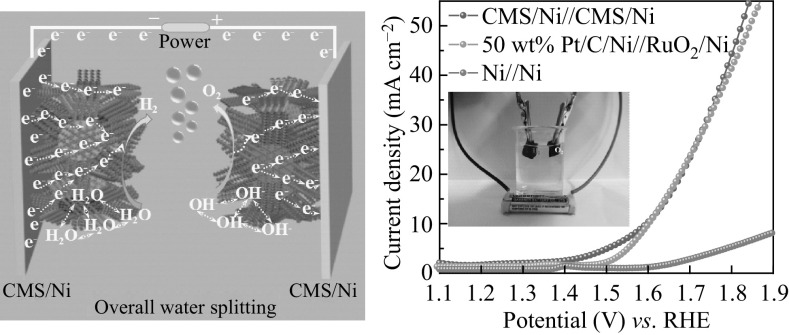

## Highlights

- Cobalt–manganese sulfides grown on Ni foam (CMS/Ni) with three-dimensionally hexagram-like nanosheets structure were prepared via a solvothermal method.
- As-prepared CMS/Ni shows highly catalytic activity for OER and HER in basic medium and can catalyze water splitting by a 1.50 V dry battery.

## Introduction

Electrocatalytic water splitting has been regarded as the most promising and feasible technology to produce clean hydrogen fuel from aqueous solutions [1–3]. Hence, efficient electrocatalysts for both the oxygen evolution reaction (OER) at anodes and hydrogen evolution reaction (HER) at cathodes are urgently needed to reduce the energy consumption for overall water splitting [4–6]. Precious metal oxide (e.g., RuO_2_, IrO_2_) and noble metal (e.g., Pt, Ir, Rh) electrocatalysts are so far known as the most efficient electrocatalysts toward OER and HER, respectively, but the high cost and scarcity have limited their widespread application [6, 7]. In this regard, tremendous efforts have been devoted to explore alternatively earth-abundant and cost-effective transition metal materials for OER or HER over the past several decades [8–22]. Unfortunately, these electrocatalysts are still not suitable for the real commercial applications. In addition, to simplify the overall water splitting system and cut the cost, developing highly efficient bifunctional electrocatalysts for both OER and HER in the same electrolyte, especially for alkaline electrolyte, has become one of the hottest issues recently [23]. Despite great advances have taken in this field [24–39], it is still in great demand to explore high-performance and non-noble bifunctional electrocatalysts for overall water splitting.

The catalytic activity could improve following the methods of chemical composition tuning and nanostructure modification [40–44]. On the one hand, to tailor the chemical composition of electrocatalysts, an effective way is doping with foreign atoms into the crystal lattice of materials. Following this way, the multicomponent or composite electrocatalysts would obtain [40–49]. The formation of different valence and electronic states of metal ions in these composites facilitates the adsorption and desorption of intermediates in the electrocatalysis process, and the synergistic effect between the metal ions is benefit for their catalytic activity. For example, Wu et al. [42] discovered the different valence states of Ni and synergistic effect between the metal ions in Ni_3_ZnC_0.7_, playing an important role in its catalytic activity for HER and OER. Yang et al. [50] synthesized a Co(II)_1−*x*_Co(0)_*x*/3_Mn(III)_2x/3_S nanoparticles combining with B/N-codoped mesoporous nanocarbon. They investigated the formation of different valence and electronic states of Co and Mn ions in facilitating the catalytic activity. On the other hand, optimizing the nanostructure of electrocatalysts can increase the quantity of effectively active sites. Comparing to nanoparticles materials, the electrocatalysts directly supported on conductive substrates, such as Ni foam, Ni mesh, Cu mesh, carbon cloth and carbon paper, with binder free can get to this point easily [41]. Recently, Ni foam has exhibited considerable potential in optimizing the nanostructure of materials [51–54]. It has a unique three-dimensional (3D) porous structure and high conductivity. For instance, our group [32] prepared urchin-like sphere arrays Co_3_O_4_ supported on 3D Ni foam showing great performance for HER and OER, which is benefit from its urchin-like nanostructure with rich mesopores and low charge-transfer resistance. Hu et al. [41] developed a Co–Mn carbonate hydroxide (CoMnCH) nanosheet arrays on Ni foam exhibiting superior activity for HER and OER in basic medium.

**Scheme 1:**
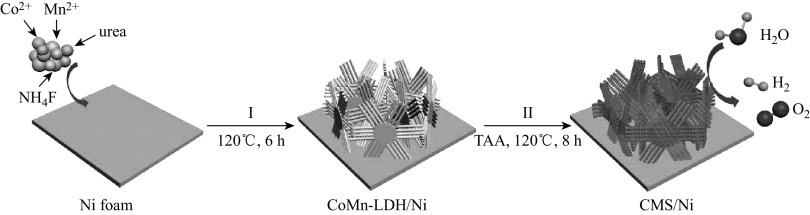
Scheme of the synthesis procedure for CMS/Ni

Based on the above mentioned, a cobalt–manganese sulfides composite with a unique nanostructure was synthesized to exhibit an efficient catalytic performance for OER and HER. However, to the best of our knowledge, the cobalt–manganese sulfide with efficient catalystic performance has never been reported. Herein, the 3D hexagram-like CMS/Ni was prepared via a simple two-step hydrothermal method (Scheme 1). Firstly, the CO_3_^2−^ and OH^−^ ions were released by the hydrolysis of urea and gradually co-precipitated with Co^2+^ and Mn^2+^ ions to form the 3D hexagram-like precursor CoMn-LDH/Ni. Subsequently, the 3D hexagram-like CMS/Ni nanosheets were synthesized after a sulfurization process employing the thioacetamide (TAA) as the sulfur sources. The electrochemical catalytic activity of this composite was evaluated by linear sweep voltammetry, electrochemical impedance spectroscopy and chronoamperometry. The electrocatalytic performance of CMS/Ni for HER and OER is optimized with respect to those of the pure Co_9_S_8_/Ni and MnS/Ni. The CMS/Ni was designed as an efficient electrolyzer for overall water splitting.

## Experimental Sections

### Materials

MnCl_2_·4H_2_O, Co(NO_3_)_2_·6H_2_O, urea and NH_4_F were purchased from Aladdin. Ni foam was purchased from Kunshan Electronic Limited Corporation. All chemicals were directly used without any purification.

### Synthesis of CoMn-LDH/Ni

Typically, 198 mg MnCl_2_·4H_2_O, 582 mg Co(NO_3_)_2_·6H_2_O, 180 mg urea and 37 mg NH_4_F were dissolved into a beaker containing 40 mL distilled water and 10 mL absolute ethanol to form a homogeneous solution under stirring for 10 min. A piece of Ni foam (3 × 3 cm^2^) which was cleaned by sonication sequentially in 3 mol L^−1^ HCl solution and absolute ethanol for 15 min each was immersed into the above solution, and then, the mixture was transferred into an autoclave (80 mL). The autoclave was sealed and heated at 120 °C for 6 h. After cooling down to room temperature, the precursor CoMn-LDH/Ni (XRD pattern is exhibited in Fig. S1) was taken out and washed by deionized water for four times and dried at 60 °C.

### Synthesis of CMS/Ni

To obtain the CMS/Ni, 400 mg thioacetamide was dissolved in 50 mL deionized water. Then, the clean solution was transferred into an autoclave containing a piece of prepared CoMn-LDH/Ni. After heating at 120 °C for 12 h, the product was taken out and severally washed by absolute ethanol and deionized water for four times. Finally, the CMS/Ni was obtained after dehydrating in an oven at 60 °C overnight. The mass loading of CMS is 4.1 mg cm^−2^. For comparison, the Co_9_S_8_/Ni and MnS/Ni were also synthesized through the same processes without adding the Mn^2+^ or Co^2+^ ions.

### Materials Characterization

The X-ray diffraction (XRD) patterns were tested by a MSAL-XD2 X-ray diffractometer with Cu Kα radiation (*λ* = 1.5406 Å). The scanning electron microscopy (SEM) was performed by a Philips SEM-XL30S microscope operated at 15 kV. High-resolution transmission electron microscope (HRTEM, JEOL JEM-2100F) coupled with an energy-dispersive X-ray spectroscopy (EDS) analyzer was carried out with an accelerating voltage of 200 kV. The nitrogen sorption isotherms were carried out by a Micromeritics TriStar 3000 Analyzer at 77 K. The X-ray photoelectron spectroscopy (XPS) was analyzed by an ESCALab250.

### Electrochemical Measurements

All the electrochemical measurements were carried out in a conventional three-electrode system. The CMS/Ni and other obtained samples were used as the working electrodes. Pt foil and Hg/HgO electrodes were separately used as the counter and reference electrodes. The 1.0 mol L^−1^ KOH was used as the electrolyte. Linear sweep voltammetry (LSV) was analyzed with a scan rate of 2 mV s^−1^. All potentials in this work were calibrated by the Nernst equation:

1 $$ E_{\text{RHE}} = E_{\text{Hg/HgO}} + (0.098 + 0.059\,{\text{pH}})\,{\text{V}} $$

2 $$ \eta = E_{\text{RHE}} - 1.23{\text{ V}} $$

where *η* is the overpotential. In addition, the Tafel slope was modeled by Tafel equation:

3 $$ \eta = b{ \log }j + a $$

where *η* is the overpotential, *b* is the Tafel slope, *j* is the current density and *a* is a constant. Chronoamperometry measurements were tested at a static potential, and the electrochemical impedance spectroscopy (EIS) was also performed in the frequency range from 10 kHz to 10 mHz with an amplitude of 5 mV. The electrochemically active surface areas (ECSAs) were evaluated by the electrochemical double-layer capacitance (*C*_dl_) via collecting cyclic voltammograms (CVs). The different CV cycles (5, 10, 15, 20, 30, and 50 mV s^−1^) were tested in the non-Faradaic potential region from 0.124 to 0.224 V *v*s RHE to determine the *C*_dl_.

## Results and Discussion

### Structure and Morphology of Materials

The XRD patterns of the as-prepared samples were performed using their powders scraped down from the Ni foam. As shown in Fig. S2a, the XRD pattern of Co_9_S_8_ exhibits the cubic crystalline phase with diffraction peaks at 29.8°, 31.1°, 47.6°, and 51.9°, which are severally corresponding to the (311), (222), (511), and (440) planes of Co_9_S_8_ (No. 02-1459). Meanwhile, the typical diffraction peaks of MnS located at 34.3° and 49.3° are matched well with the (200) and (220) planes of cubic MnS (No. 65-2919). Interestingly, the XRD pattern of CMS reveals the crystal structures of the Co_9_S_8_ and MnS are still maintained in CMS after modulating with them as a composite. Moreover, the EDS analysis reveals the atomic ratio of Co, Mn and S is ~0.9:0.1:1.0 in the composite (Fig. S2b), while the Cu elemental is coming from copper mesh. Figure S2c, d shows the atomic ratios of Co_9_S_8_ and MnS are ~9.0:8.0 and 1.0:1.0, respectively.

**Fig. 1:**
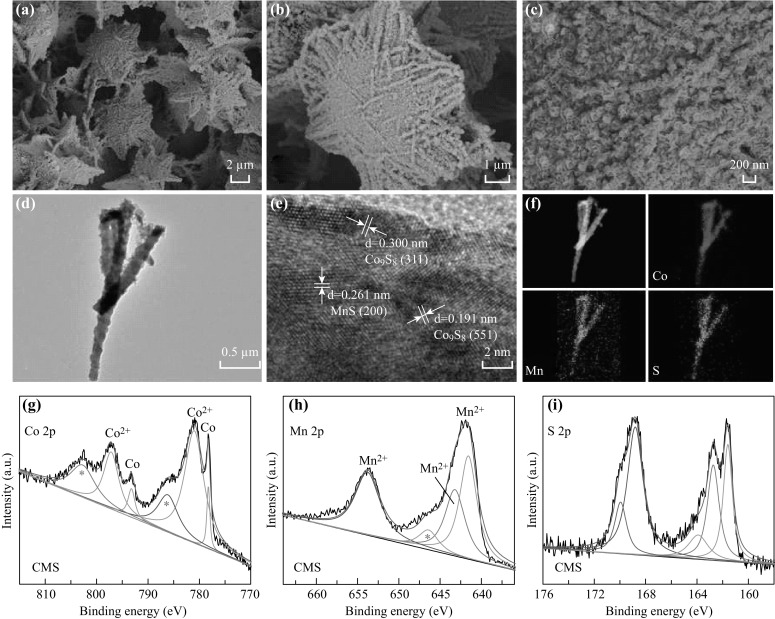
The characterization results of CMS/Ni: **a**–**c** SEM images in different magnifications, **d** TEM image, **e** HRTEM image, **f** elemental mapping images and **g**–**i** XPS spectra

Figure S3 presents the SEM images of Co_9_S_8_/Ni and MnS/Ni. The nanosheet structures of Co_9_S_8_/Ni and MnS/Ni are still retained a rough surface with respect to their precursors. However, the morphologies are modified after combining with Co_9_S_8_/Ni and MnS/Ni. It can be observed in Fig. 1a, b that the 3D hexagram-like CMS/Ni nanosheets were obtained after sulfonating the CoMn-LDH/Ni (Fig. S4), and each “hexagram” is entirely covered by uniform nanosheets (Fig. 1c). The TEM image shown in Fig. 1d reveals the 3D hexagram-like CMS/Ni nanosheets which are made up of solid nanorods. Figure 1e shows the HRTEM image, where the distinct lattice fringes of *d* = 0.300 nm and *d* = 0.191 nm are corresponding to (311) and (511) crystal plane of Co_9_S_8_, respectively, and the *d* = 0.261 nm is attributed to (200) crystal plane of MnS. This result indicates that the CMS is a composite material, which is comprised by the interrelated Co_9_S_8_ and MnS. Additionally, it can be seen that the elements of Co, Mn and S are uniformly dispersed in CMS/Ni, as shown in Fig. 1f.

The Co 2p XPS spectra of CMS/Ni in Fig. 1g reveal two distinct peaks at 781.0 and 797.3 eV corresponding to the Co 2*p*_3/2_ and Co 2*p*_1/2_, respectively, with two related satellite peaks at 786.3 and 802.9 eV. These are the characteristic peaks of Co^2+^ [50], and the other two peaks at 778.2 and 793.2 eV are assigned to the metallic Co [50]. Nevertheless, there is not metallic Co presented on the Co 2*p* XPS spectra of Co_9_S_8_/Ni counterpart (Fig. S5a). Moreover, there are no diffraction peaks of metallic Co presenting in the XRD pattern of CMS/Ni, which may be attributed to its low content in CMS/Ni. As Mn 2*p* XPS spectra of CMS/Ni shown in Fig. 1h, the peak located at 643.3 eV confirms the oxidized Mn^3+^ species in CMS/Ni [50, 55], while there is no Mn^3+^ detected from XPS spectra of MnS/Ni counterpart (Fig. S5b). Simultaneously, Mn^2+^ reveals with two characteristic peaks at bending energies of 641.6 eV (Mn 2*p*_3/2_) and 653.8 eV (Mn 2*p*_1/2_). The above results are similar to Yang’s work [50]. The occurrence of metallic Co and Mn^3+^ in CMS/Ni can be explained by the higher reduction potential of Co^2+^/Co (Co^2+^ + 2e^−^ → Co, − 0.277 V vs. NHE) comparing to Mn^2+^/Mn (Mn^2+^ + 2e^−^ → Mn, − 1.18 V vs. NHE). This means that the Co^2+^ as oxidizer is easier to be reduced from Co^2+^ to metallic Co than the same reaction of Mn^2+^. The reduction in Co^2+^ to metallic Co would result in the oxidation of Mn^2+^ to Mn^3+^ at the same time (Mn^2+^ − e^−^ → Mn^3+^, 1.5 V vs. NHE) [56]. The different valance states of metal cations in CMS/Ni are benefit for improving the catalytic performance [42, 50]. Figure 1i shows the peaks of S 2*p*_3/2_ and S 2*p*_1/2_ are located at 161.6 and 162.7 eV, respectively, which are derived from metal–sulfur bonds [50]. Furthermore, a peak at 168.8 eV with its satellite peak at 170.0 eV is attributed to the superficial oxidation of CMS/Ni in air [55].

### Hydrogen Evolution Activity

**Fig. 2:**
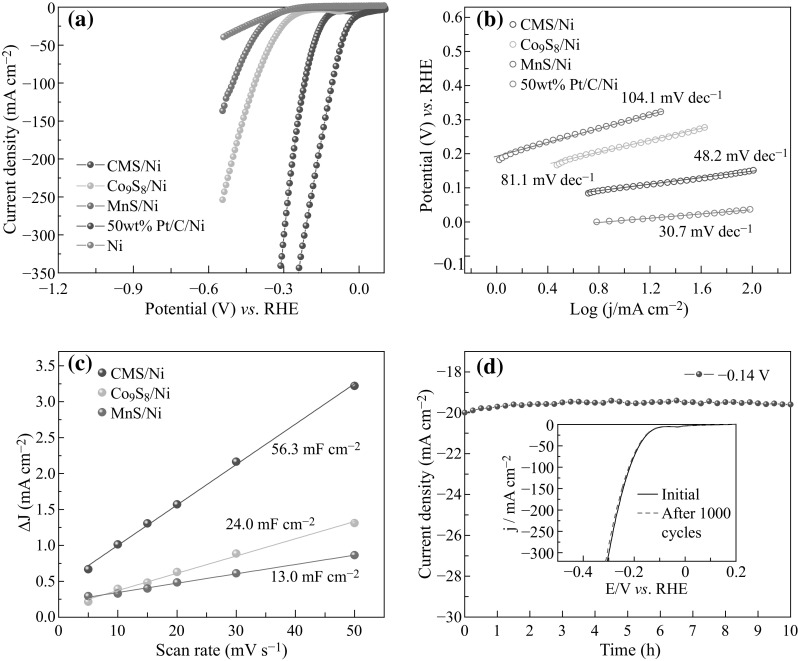
**a** Polarization curves, **b** Tafel plots, **c** plots of the current density for CMS/Ni, Co_9_S_8_/Ni, MnS/Ni at potential of 0.174 V against the scan rates and **d** chronoamperometric curve (the inset is polarization curves before and after 1000 CV cycles)

The electrocatalytic activity of CMS/Ni toward HER was characterized by LSV measurement with a scan rate of 2 mV s^−1^. For comparison, the Co_9_S_8_/Ni, MnS/Ni, bare Ni foam and commercial 50 wt% Pt/C coated on Ni foam (50 wt% Pt/C/Ni [30], loading 4.1 mg cm^−2^) were also evaluated. As the polarization curves illustrated in Fig. 2a, the 50 wt% Pt/C/Ni possesses the most excellent HER performance with a near-zero onset potential. The CMS/Ni reveals a much smaller onset potential at − 88 mV and larger HER current than those of Co_9_S_8_/Ni, MnS/Ni and bare Ni foam. This result highlights the catalytic activity of CMS/Ni has significantly enhanced after combining Co_9_S_8_/Ni with MnS/Ni as a composite, which may be ascribed to the synergistic effect of Co and Mn. The HER performance of CMS/Ni is also comparable with the CoMn-LDH/Ni, as shown in Fig. S6a, b. Noticeably, the CMS/Ni drives a high current density of 100 mA cm^−2^ at an overpotential of 217 mV, which is lower than 419 mV for Co_9_S_8_/Ni and 506 mV for MnS/Ni. Such an overpotential of CMS/Ni is superior to most of the recently reported bifunctional electrocatalysts listed in Table S1. The efficient catalytic activity of CMS/Ni is also supported by the Tafel slopes in Fig. 2b. The Tafel slope of CMS/Ni is 48.2 mV dec^−1^ which is obviously lower than 81.1 mV dec^−1^ for Co_9_S_8_/Ni and 104.1 mV dec^−1^ for MnS/Ni, implying great HER kinetics and catalytic activity. In addition, the EIS measurements of CMS/Ni, Co_9_S_8_/Ni and MnS/Ni were measured at a static potential of − 0.33 V to further elucidate the charge transport of the as-prepared materials. As exhibited in Fig. S7, the CMS/Ni shows lower resistance of 0.98 Ω than the pure Co_9_S_8_/Ni (1.80 Ω) and MnS/Ni (2.14 Ω), suggesting an improvement of conductivity. The lower charge-transfer resistance of CMS/Ni may be attributed to its uniquely 3D hexagram-like nanosheets structure contacting with the electrolyte efficiently, and the metallic Co generated in CMS (see Fig. 1g) after integrating with two Co_9_S_8_ and MnS to be the composite [50].

On the other hand, the superior activity of CMS/Ni for HER, in comparison with that of the pure Co_9_S_8_/Ni and MnS/Ni, results from the significant increase in electrochemical active surface areas (ECSAs) to expose more accessible catalytic active sites. The ECSAs of CMS/Ni, Co_9_S_8_/Ni and MnS/Ni were measured by the capacitance measurements through cyclic voltammograms in a non-Faradaic at different scan rates (Fig. S8). The ECSA of an electrocatalyst is proportional to its *C*_dl_ value. It can be seen that the *C*_dl_ values of 24.0 mF cm^−2^ for Co_9_S_8_/Ni and 13.0 mF cm^−2^ for MnS/Ni are tremendously increase to 56.3 mF cm^−2^ for CMS/Ni, implying the CMS/Ni has more effective active sites, as shown in Fig. 2c.

The HER stability of CMS/Ni was further evaluated at a constant potential of − 0.14 V. As shown in Fig. 2d, the CMS/Ni reveals a great stability with a negligible decay of the current density after 10 h continuous measurements. Simultaneously, the LSV polarization curve tested at 100 mV s^−1^ after 1000 cycles is similar to the first cycle, as the inset in Fig. 2d. The superior durability of CMS/Ni is benefit from its high structure stability because the hexagram-like structure for CMS/Ni just exhibits a little aggregation after stability measurement in Fig. S9a. The excellent catalytic performance and great stability highlight the great potential of CMS/Ni for practical application.

### Oxygen Evolution Activity

**Fig. 3:**
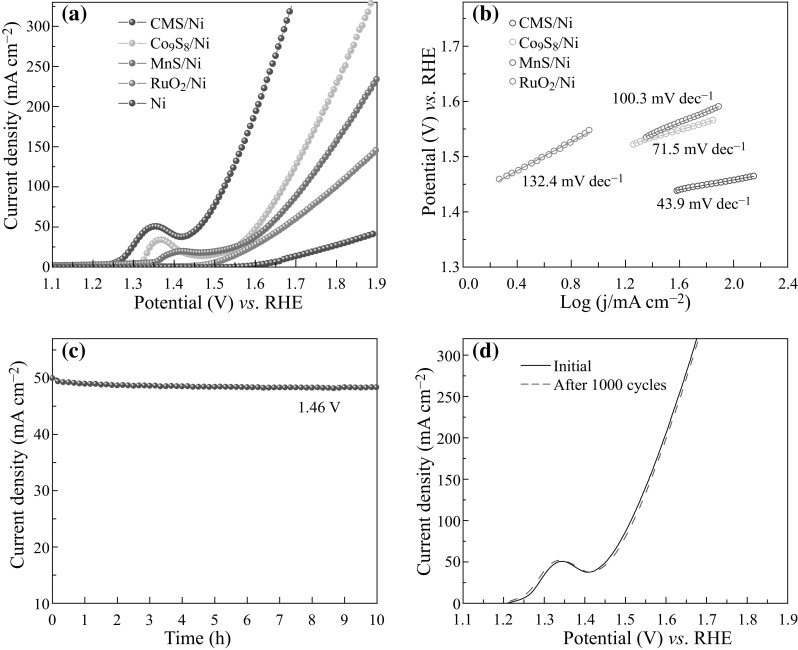
**a** Polarization curves, **b** Tafel plots, **c** chronoamperometric curve and **d** polarization curves before and after 1000 CV cycles

The electrocatalytic activity of CMS/Ni for OER was also characterized by LSV measurement with a scan rate of 2 mV s^−1^. The Co_9_S_8_/Ni, MnS/Ni, bare Ni foam and commercial RuO_2_ coated on Ni foam (RuO_2_/Ni [30], loading 4.1 mg cm^−2^) were evaluated for comparison. Figure 3a shows the polarization curves of the as-prepared samples, in which the oxidation peaks from 1.33 to 1.41 V are ascribed to the transition from M^2+^ to M^3+^ [30]. Owing to the intense oxidation peaks, the overpotential for materials to generate the anodic current of 10 mA cm^−2^ for OER is not accurate. Therefore, we report the overpotentials at 100 mA cm^−2^ here. As observed, a current density of 100 mA cm^−2^ is easily reached at the overpotential of 298 mV for CMS/Ni, better than those of Co_9_S_8_/Ni (439 mV), MnS/Ni (492 mV), RuO_2_/Ni (568 mV) and even most of the other recently reported bifunctional materials exhibited in Table S1. The above results suggest the efficient OER activity of CMS/Ni, which is also superior to the CoMn-LDH/Ni (Fig. S6c, d). A tremendous improvement of catalytic performance for CMS/Ni with respect to its monometallic counterparts can also be supported by its smallest Tafel slope. As shown in Fig. 3b, the smallest Tafel slopes of 43.9 mV dec^−1^ for CMS/Ni with respect to Co_9_S_8_/Ni (71.5 mV dec^−1^), MnS/Ni (100.3 mV dec^−1^) and RuO_2_/Ni (132.4 mV dec^−1^) imply the great OER kinetics activity.

The stability of CMS/Ni for OER was calculated at a static potential of 1.46 V in 1.0 mol L^−1^ KOH solution. Figure 3c presents a negligible decrease in current density after 10 h continuing OER measurement, indicating the superior durability. Additionally, this result can be further confirmed by the LSV curves in Fig. 3d, because the polarization curve after 1000 cycles is similar to the initial cycle. In particular, it can be seen that the morphology of CMS/Ni slightly changes after stability testing in Fig. S9b.

All the above results indicate that the CMS/Ni possesses superior HER and OER catalytic activity comparing to the pure Co_9_S_8_/Ni and MnS/Ni, which could be involved the following factors: (1) The obtained CMS/Ni has a uniquely 3D hexagram-like nanosheet structure, which not only provides a large electrochemical active surface areas (ECSAs) to expose more accessible catalytic active sites, but also contacts with the electrolyte efficiently and facilitates the transportation of O_2_, H_2_ bubbles [28, 32, 57]; (2) the 3D hexagram-like CMS directly supported on Ni foam substrate enhances the structure stability and improves electrons transport ability of CMS/Ni, which are beneficial to improving the catalytic activity; and (3) the XPS results (shown in Fig. 1g, h) indicate that the different valence states of Co and Mn are presented in the CMS/Ni, which can facilitate the adsorption and desorption of intermediates in the electrocatalysis process. The synergistic effect between Co and Mn is helpful for the catalytic activity [42, 49, 50, 58].

### Overall Water Splitting

**Fig. 4:**
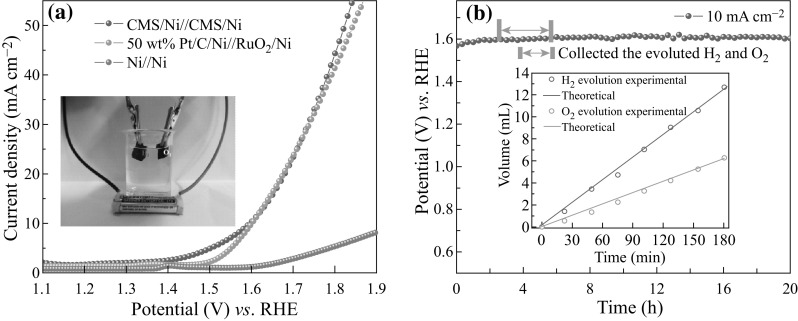
**a** Polarization curves of CMS/Ni//CMS/Ni, 50 wt Pt/C/Ni//RuO_2_/Ni and Ni//Ni for overall water splitting (the inset is a device for overall water splitting). **b** Chronopotentiometry curve of CMS/Ni//CMS/Ni (the inset is volume of O_2_ and H_2_ generated for theoretically calculated and experimentally measured versus time)

Based on the excellent activity toward OER and HER, the CMS/Ni was directly assembled as the cathodic and anodic electrodes in two-electrode cell (CMS/Ni//CMS/Ni) in 1.0 mol L^−1^ KOH electrolyte for overall water splitting. As shown in Fig. 4a, the LSV curve of the cell indicates that water is electrolyzed only at the voltage of 1.47 V to generate H_2_ and O_2_ bubbles. This result is supported by a device, which is driven by a 1.50 V dry battery (the inset in Figs. 4a and S10). For comparison, the 50 wt% Pt/C/Ni, RuO_2_/Ni and bare Ni foam were also used as the two-electrode electrolyzers. A current density of 10 mA cm^−2^ for CMS/Ni//CMS/Ni can be achieved at a cell voltage of 1.60 V, equaling to that of 1.60 V for 50 wt% Pt/C/Ni//RuO_2_/Ni. Although this cell voltage (1.60 V) of CMS/Ni//CMS/Ni is higher than 1.52 V for Cu@CoS_*x*_/CF–Cu@CoS_*x*_/CF [59], 1.45 V for MoO_*x*_/Ni_3_S_2_/NF//MoO_*x*_/Ni_3_S_2_/NF [60] and 1.53 V for NixCo_3−*x*_S_4_/Ni_3_S_2_/NF//Ni_*x*_Co_3−*x*_S_4_/Ni_3_S_2_/NF [61], it is superior to that of NiCo_2_S_4_ NW/NF//NiCo_2_S_4_ NW/NF (1.68 V) [25], FeNi_3_N/NF//FeNi_3_N/NF (1.62 V) [27], Ni/NiP//Ni/NiP (1.61 V) [30], Co_3_O_4_@Ni//Co_3_O_4_@Ni (1.64 V) [32], Ni_2.5_Co_0.5_Fe/NF//Ni_2.5_Co_0.5_Fe/NF (1.62 V) [35], Ni(OH)_2_/NF//Ni(OH)_2_/NF (1.68 V) [36], Co_1_Mn_1_CH/NF//Co_1_Mn_1_CH/NF (1.68 V) [41], Co(S_0.71_Se_0.29_)_2_//Co(S_0.22_Se_0.78_)_2_ (1.63 V) [43] and the two-electrode electrolyzers listed in Table S1. In addition, the chronopotentiometry curve of CMS/Ni//CMS/Ni was measured at a constant current of 10 mA cm^−2^ for 20 h, exhibiting a great stability for CMS/Ni//CMS/Ni in Fig. 4b. The volume–time plots for generating O_2_ and H_2_ suggest that the Faradaic efficiency of CMS/Ni//CMS/Ni electrolyzer is nearly 100% (see the inset in Fig. 4b and experimental details are shown in SI).

## Conclusions

In summary, a simple anion exchange method was employed to successfully prepare 3D hexagram-like CMS/Ni. The 3D hexagram-like CMS/Ni nanosheets have large electrochemical active surface area to expose more active sites and low charge-transfer resistance. Noticeably, the synergetic effect of Co and Mn is also presented in this composite. Consequently, it exhibits superior catalytic activity in basic medium with low overpotentials of 217 mV for HER and 298 mV for OER to reach a current density of 100 mA cm^−2^. More importantly, the assembled CMS/Ni//CMS/Ni device for overall water splitting can be driven by a 1.50 V dry battery, indicating the great potential for practical applications. Therefore, this work provides a scalable method to synthesize bi- or multimetallic sulfide composites and extends the preparation of the other novel electrocatalysts for water splitting.

## Electronic supplementary material

Below is the link to the electronic supplementary material.

Supplementary material 1 (PDF 1405 kb)

## References

[1] Morales-Guio CG, Mayer MT, Yella A, Tilley SD, Gratzel M, Hu X (2015). An optically transparent iron nickel oxide catalyst for solar water splitting. J. Am. Chem. Soc..

[2] Walter MG, Warren EL, McKone JR, Boettcher SW, Mi Q, Santori EA, Lewis NS (2010). Solar water splitting cells. Chem. Rev..

[3] Kuai L, Geng J, Chen C, Kan E, Liu Y, Wang Q, Geng B (2014). A reliable aerosol-spray-assisted approach to produce and optimize amorphous metal oxide catalysts for electrochemical water splitting. Angew. Chem. Int. Ed..

[4] Suntivich J, May KJ, Gasteiger HA, Goodenough JB, Shao-Horn Y (2011). A perovskite oxide optimized for oxygen evolution catalysis from molecular orbital principles. Science.

[5] Rausch B, Symes MD, Chisholm G, Cronin L (2014). Decoupled catalytic hydrogen evolution from a molecular metal oxide redox mediator in water splitting. Science.

[6] Ng JWD, García-Melchor M, Bajdich M, Chakthranont P, Kirk C, Vojvodic A, Jaramillo TF (2016). Gold-supported cerium-doped NiO_*x*_ catalysts for water oxidation. Nat. Energy.

[7] Gorlin Y, Jaramillo TF (2010). A bifunctional nonprecious metal catalyst for oxygen reduction and water oxidation. J. Am. Chem. Soc..

[8] Zhang X, Si C, Guo X, Konga R, Qu F (2017). MnCo_2_S_4_ nanowire array as an earth-abundant electrocatalyst for efficient oxygen evolution reaction under alkaline conditions. J. Mater. Chem. A.

[9] Zhang J, Hu Y, Liu D, Yu Y, Zhang B (2017). Enhancing oxygen evolution reaction at high current densities on amorphous-like Ni–Fe–S ultrathin nanosheets via oxygen incorporation and electrochemical tuning. Adv. Sci..

[10] Yang D, Gao L, Yang J-H (2017). Facile synthesis of ultrathin Ni(OH)_2_–Cu_2_S hexagonal nanosheets hybrid for oxygen evolution reaction. J. Power Sources.

[11] Long J, Gong Y, Lin J (2017). Metal-organic framework-derived Co_9_S_8_@CoS@CoO@C nanoparticles as efficient electro- and photo-catalysts for the oxygen evolution reaction. J. Mater. Chem. A.

[12] Guan BY, Yu L, Lou XW (2017). General synthesis of multishell mixed-metal oxyphosphide particles with enhanced electrocatalytic activity in the oxygen evolution reaction. Angew. Chem. Int. Ed..

[13] Suen NT, Hung SF, Quan Q, Zhang N, Xu YJ, Chen HM (2017). Electrocatalysis for the oxygen evolution reaction: recent development and future perspectives. Chem. Soc. Rev..

[14] Chen X, Zhang Z, Chi L, Nair AK, Shangguan W, Jiang Z (2015). Recent advances in visible-light-driven photoelectrochemical water splitting: catalyst nanostructures and reaction systems. Nano-Micro Lett..

[15] Li R, Yang L, Xiong T, Wu Y, Cao L, Yuan D, Zhou W (2017). Nitrogen doped MoS_2_ nanosheets synthesized via a low-temperature process as electrocatalysts with enhanced activity for hydrogen evolution reaction. J. Power Sources.

[16] Wang C, Tian B, Wu M, Wang J (2017). Revelation of the excellent intrinsic activity of MoS_2_|NiS|MoO_3_ nanowires for hydrogen evolution reaction in alkaline medium. ACS Appl. Mater. Interfaces.

[17] Wu Z, Guo J, Wang J, Liu R, Xiao W, Xuan C, Xia K, Wang D (2017). Hierarchically porous electrocatalyst with vertically aligned defect-rich CoMoS nanosheets for the hydrogen evolution reaction in an alkaline medium. ACS Appl. Mater. Interfaces.

[18] Wang N, Hang T, Chu D, Li M (2015). Three-dimensional hierarchical nanostructured Cu/Ni–Co coating electrode for hydrogen evolution reaction in alkaline media. Nano-Micro Lett..

[19] Li X, Liu PF, Zhang L, Zu MY, Yang YX, Yang HG (2016). Enhancing alkaline hydrogen evolution reaction activity through Ni–Mn_3_O_4_ nanocomposites. Chem. Commun..

[20] Li Q, Wang F, Sun L, Jiang Z, Ye T, Chen M, Bai Q, Wang C, Han X (2017). Design and synthesis of Cu@CuS yolk-shell structures with enhanced photocatalytic activity. Nano-Micro Lett..

[21] Yan X, Tian L, Murowchick J, Chen X (2016). Partially amorphized MnMoO_4_ for highly efficient energy storage and the hydrogen evolution reaction. J. Mater. Chem. A.

[22] Shao L, Qian X, Wang X, Li H, Yan R, Hou L (2016). Low-cost and highly efficient CoMoS_4_/NiMoS_4_-based electrocatalysts for hydrogen evolution reactions over a wide pH range. Electrochim. Acta.

[23] Luo J, Im J-H, Mayer MT, Schreier M, Nazeeruddin MK, Park N-G, Tilley SD, Fan HJ, Grätzel M (2014). Water photolysis at 12.3% efficiency via perovskite photovoltaics and Earth-abundant catalysts. Science.

[24] Xing J, Li H, Ming-Cheng Cheng M, Geyer SM, Ng KYS (2016). Electro-synthesis of 3D porous hierarchical Ni–Fe phosphate film/Ni foam as a high-efficiency bifunctional electrocatalyst for overall water splitting. J. Mater. Chem. A.

[25] Sivanantham A, Ganesan P, Shanmugam S (2016). Hierarchical NiCo_2_S_4_ nanowire arrays supported on Ni foam: an efficient and durable bifunctional electrocatalyst for oxygen and hydrogen evolution reactions. Adv. Funct. Mater..

[26] Liu D, Lu Q, Luo Y, Sun X, Asiri AM (2015). NiCo_2_S_4_ nanowires array as an efficient bifunctional electrocatalyst for full water splitting with superior activity. Nanoscale.

[27] Zhang B, Xiao C, Xie S, Liang J, Chen X, Tang Y (2016). Iron-nickel nitride nanostructures in situ grown on surface-redox-etching nickel foam: efficient and ultrasustainable electrocatalysts for overall water splitting. Chem. Mater..

[28] You B, Jiang N, Sheng M, Bhushan MW, Sun Y (2016). Hierarchically porous urchin-like Ni_2_P superstructures supported on nickel foam as efficient bifunctional electrocatalysts for overall water splitting. ACS Catal..

[29] Feng L-L, Yu G, Wu Y, Li G-D, Li H, Sun Y, Asefa T, Chen W, Zou X (2015). High-index faceted Ni_3_S_2_ nanosheet arrays as highly active and ultrastable electrocatalysts for water splitting. J. Am. Chem. Soc..

[30] Chen G-F, Ma TY, Liu Z-Q, Li N, Su Y-Z, Davey K, Qiao S-Z (2016). Efficient and stable bifunctional electrocatalysts Ni/NixMy (M = P, S) for overall water splitting. Adv. Funct. Mater..

[31] Tang C, Cheng N, Pu Z, Xing W, Sun X (2015). NiSe Nanowire film supported on nickel foam: an efficient and stable 3D bifunctional electrode for full water splitting. Angew. Chem. Int. Ed..

[32] Li R, Zhou D, Luo J, Xu W, Li J, Li S, Cheng P, Yuan D (2017). The urchin-like sphere arrays Co_3_O_4_ as a bifunctional catalyst for hydrogen evolution reaction and oxygen evolution reaction. J. Power Sources.

[33] Zhu W, Yue X, Zhang W, Yu S, Zhang Y, Wang J, Wang J (2016). Nickel sulfide microsphere film on Ni foam as an efficient bifunctional electrocatalyst for overall water splitting. Chem. Commun..

[34] Xu R, Wu R, Shi Y, Zhang J, Zhang B (2016). Ni_3_Se_2_ nanoforest/Ni foam as a hydrophilic, metallic, and self-supported bifunctional electrocatalyst for both H_2_ and O_2_ generations. Nano Energy.

[35] Zhu X, Tang C, Wang H-F, Li B-Q, Zhang Q, Li C, Yang C, Wei F (2016). Monolithic-structured ternary hydroxides as freestanding bifunctional electrocatalysts for overall water splitting. J. Mater. Chem. A.

[36] Rao Y, Wang Y, Ning H, Li P, Wu M (2016). Hydrotalcite-like Ni(OH)_2_ nanosheets in situ grown on nickel foam for overall water splitting. ACS Appl. Mater. Interfaces.

[37] Wang Z, Zeng S, Liu W, Wang X, Li Q, Zhao Z, Geng F (2017). Coupling molecularly ultrathin sheets of NiFe-layered double hydroxide on NiCo_2_O_4_ nanowire arrays for highly efficient overall water-splitting activity. ACS Appl. Mater. Interfaces.

[38] Yang Y, Zhang K, Lin H, Li X, Chan HC, Yang L, Gao Q (2017). MoS_2_–Ni_3_S_2_ heteronanorods as efficient and stable bifunctional electrocatalysts for overall water splitting. ACS Catal..

[39] Miao R, He J, Sahoo S, Luo Z, Zhong W (2017). Reduced graphene oxide supported nickel-manganese-cobalt spinel ternary oxide nanocomposites and their chemically converted sulfide nanocomposites as efficient electrocatalysts for alkaline water splitting. ACS Catal..

[40] Li J, Wei G, Zhu Y, Xi Y, Pan X, Ji Y, Zatovsky IV, Han W (2017). Hierarchical NiCoP nanocone arrays supported on Ni foam as an efficient and stable bifunctional electrocatalyst for overall water splitting. J. Mater. Chem. A.

[41] Tang T, Jiang W-J, Niu S, Liu N, Luo H (2017). Electronic and morphological dual modulation of cobalt carbonate hydroxides by Mn doping toward highly efficient and stable bifunctional electrocatalysts for overall water splitting. J. Am. Chem. Soc..

[42] Wang Y, Wu W, Rao Y, Li Z, Tsubaki N, Wu M (2017). Cation modulating electrocatalyst derived from bimetallic metal-organic frameworks for overall water splitting. J. Mater. Chem. A.

[43] Fang L, Li W, Guan Y, Feng Y, Zhang H, Wang S, Wang Y (2017). Tuning unique peapod-like Co(S_*x*_Se_1−*x*_)_2_ nanoparticles for efficient overall water splitting. Adv. Funct. Mater..

[44] Wang X-D, Chen H-Y, Xu Y-F, Liao J-F, Chen B-X, Rao H-S, Kuang D-B, Su C-Y (2017). Self-supported NiMoP_2_ nanowires on carbon cloth as an efficient and durable electrocatalyst for overall water splitting. J. Mater. Chem. A.

[45] Li J, Xu W, Li R, Luo J, Zhou D, Li S, Cheng P, Yuan D (2016). A tremella-like Ni_76_Co_24_ layered double hydroxides nanosheets as an efficient catalyst for oxygen evolution reaction. J. Mater. Sci..

[46] Yang Y, Lin Z, Gao S, Su J, Lun Z, Xia G, Chen J, Zhang R, Chen Q (2017). Tuning electronic structures of nonprecious ternary alloys encapsulated in graphene layers for optimizing overall water splitting activity. ACS Catal..

[47] Fan X, Peng Z, Ye R, Zhou H, Guo X (2015). M3C (M: Fe Co, Ni) nanocrystals encased in graphene nanoribbons: an active and stable bifunctional electrocatalyst for oxygen reduction and hydrogen evolution reactions. ACS Nano.

[48] Xu J, Cui J, Guo C, Zhao Z, Jiang R (2016). Ultrasmall Cu_7_S_4_@MoS_2_ hetero-nanoframes with abundant active edge sites for ultrahigh-performance hydrogen evolution. Angew. Chem. Int. Ed..

[49] Wang A-L, Lin J, Xu H, Tong Y-X, Li G-R (2016). Ni_2_P–CoP hybrid nanosheet arrays supported on carbon cloth as an efficient flexible cathode for hydrogen evolution. J. Mater. Chem. A.

[50] Wang Z, Xiao S, An Y, Long X, Zheng X, Lu X, Tong Y, Yang S (2016). Co(II)_1−*x*_Co(0)_*x*/3_Mn(III)_2*x*/3_S nanoparticles supported on B/N-codoped mesoporous nanocarbon as a bifunctional electrocatalyst of oxygen reduction/evolution for high-performance zinc-air batteries. ACS Appl. Mater. Interfaces.

[51] Chen P, Zhou T, Zhang M, Tong Y, Zhong C, Zhang N, Zhang L, Wu C, Xie Y (2017). 3D nitrogen-anion-decorated nickel sulfides for highly efficient overall water splitting. Adv. Mater..

[52] Wang Y, Liu D, Liu Z, Xie C, Huo J, Wang S (2016). Porous cobalt–iron nitride nanowires as excellent bifunctional electrocatalysts for overall water splitting. Chem. Commun..

[53] Ming F, Liang H, Shi H, Xu X, Mei G, Wang Z (2016). MOF-derived Co-doped nickel selenide/C electrocatalysts supported on Ni foam for overall water splitting. J. Mater. Chem. A.

[54] Jin Y, Yue X, Shu C, Huang S, Shen PK (2017). Three-dimensional porous MoNi_4_ networks constructed by nanosheets as bifunctional electrocatalysts for overall water splitting. J. Mater. Chem. A.

[55] Li Q, Xing Z, Wang D, Sun X, Yang X (2016). In situ electrochemically activated CoMn-S@NiO/CC nanosheets array for enhanced hydrogen evolution. ACS Catal..

[56] Jana SK, Saha B, Satpati B, Banerjee S (2015). Structural and electrochemical analysis of a novel co-electrodeposited Mn_2_O_3_–Au nanocomposite thin film. Dalton Trans..

[57] Li S, Wang P, Cheng J, Luo D, Zhou W, Xu J, Li R, Li D Yuan (2017). High-performance flexible asymmetric supercapacitor based on CoAl-LDH and rGO electrodes. Nano-Micro Lett..

[58] Stamenkovic VR, Strmcnik D, Lopes PP, Markovic NM (2016). Energy and fuels from electrochemical interfaces. Nat. Mater..

[59] Liu Y, Li Q, Si R, Li G-D, Li W (2017). Coupling sub-nanometric copper clusters with quasi amorphous cobalt sulfide yields efficient and robust electrocatalysts for water splitting reaction. Adv. Mater..

[60] Wu Y, Li G-D, Liu Y, Yang L, Lian X, Asefa T, Zou X (2016). Overall water splitting catalyzed efficiently by an ultrathin nanosheet-built, hollow Ni_3_S_2_-based electrocatalyst. Adv. Funct. Mater..

[61] Wu Y, Liu Y, Li G-D, Zou X, Lian X, Wang D, Sun L, Asefa T, Zou X (2017). Efficient electrocatalysis of overall water splitting by ultrasmall NixCo_3−*x*_S_4_ coupled Ni_3_S_2_ nanosheet arrays. Nano Energy.

