# Supplementary material for: Synthesis of 3D Hexagram-Like Cobalt–Manganese Sulfides Nanosheets Grown on Nickel Foam: A Bifunctional Electrocatalyst for Overall Water Splitting
Source: Nanomicro Lett. 2017 Oct 13;10(1):6. doi: 10.1007/s40820-017-0160-6 (PMC6199054; doi:10.1007/s40820-017-0160-6)
Supplement: Supplementary file 1 — Supplementary material 1 (PDF 1405 kb) [file 40820_2017_160_MOESM1_ESM.pdf]

Supporting Information for

Synthesis of 3D Hexagram-like Cobalt-Manganese Sulfides Nanosheets Grown on Nickel Foam: A Bifunctional Electrocatalyst for Overall Water Splitting

Jingwei Li[1], Weiming Xu[1], Jiaxian Luo[1], Dan Zhou[1], Dawei Zhang[1], Licheng Wei[1], Peiman Xu[1], Dingsheng Yuan[1, *]

[1]School of Chemistry and Materials Science, Jinan University, Guangzhou 510632, People's Republic of China

* Corresponding author. E-mail: tydsh@jnu.edu.cn

1. Faradic Efficiency

Two-electrode water electrolysis was operated by chronopotentiometry measurement at a constant current of 10 mA cm^{-2}. 1.0 mol L^{-1} KOH solution was used as the electrolyte. The oxygen and hydrogen bubbles were collected by a water splitting apparatus continuing for 180 min. The theoretical volume of O_2 and H_2 were calculated by the following method,

$$V_{O2}\ mL = Q\ C \times 22.4\ L\ mol^{-1} \times 1000 / (F\ C\ mol^{-1} \times 4)$$

$$V_{H2}\ mL = Q\ C \times 22.4\ L\ mol^{-1} \times 1000 / (F\ C\ mol^{-1} \times 2)$$

where Q is the cumulative charge (C), F is the Faraday constant (C mol^{-1}) [1].

2. Figures

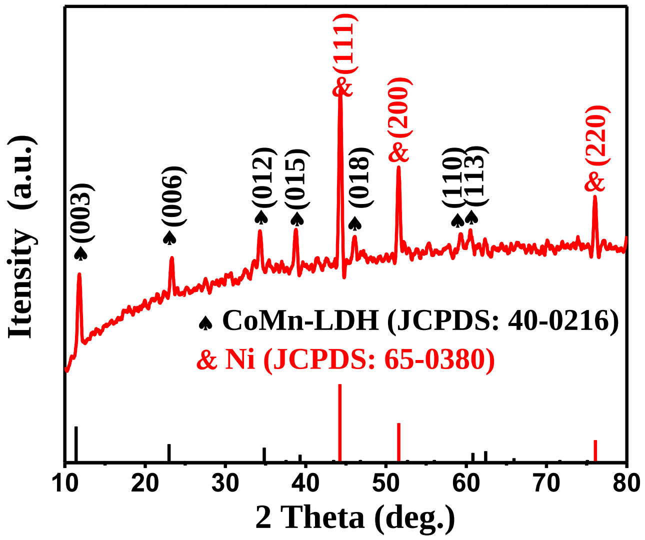

Fig. S1 XRD pattern of CoMn-LDH/Ni

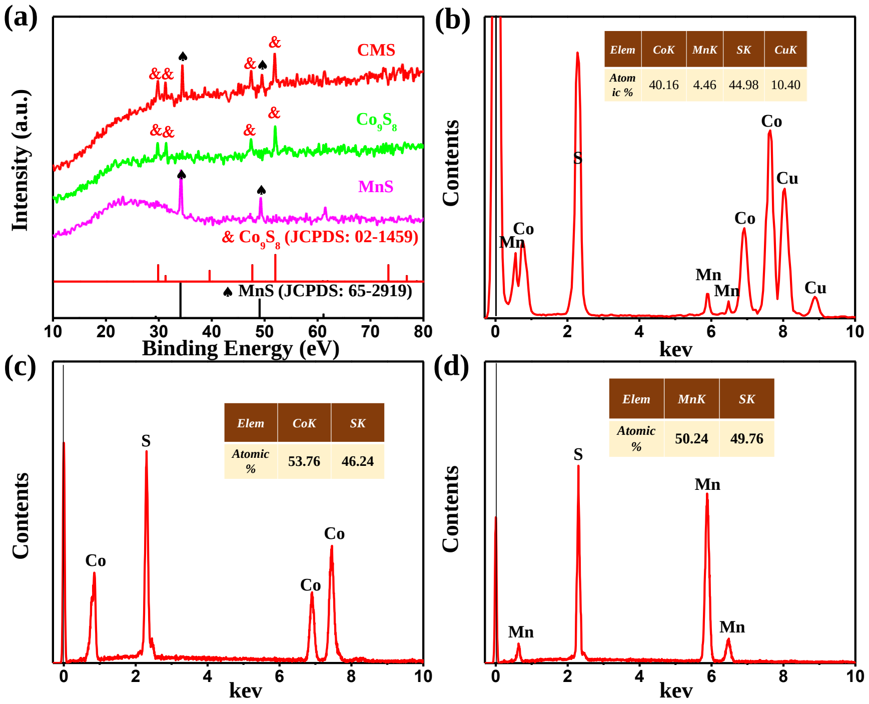

Fig. S2 a XRD patterns of CMS/Ni, Co_9S_8/Ni, and MnS/Ni. **b-d** EDS patterns of CMS/Ni, Co_9S_8/Ni, and MnS/Ni, respectively

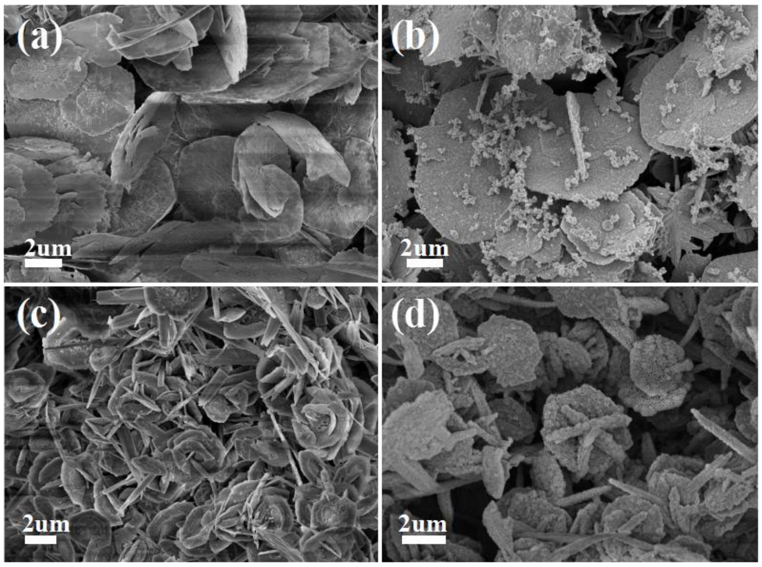

Fig. S3 SEM image for the precursor of **a** Co_9S_8/Ni and **b** Co_9S_8/Ni. SEM images for the precursor of **c** MnS/Ni and **d** MnS/Ni

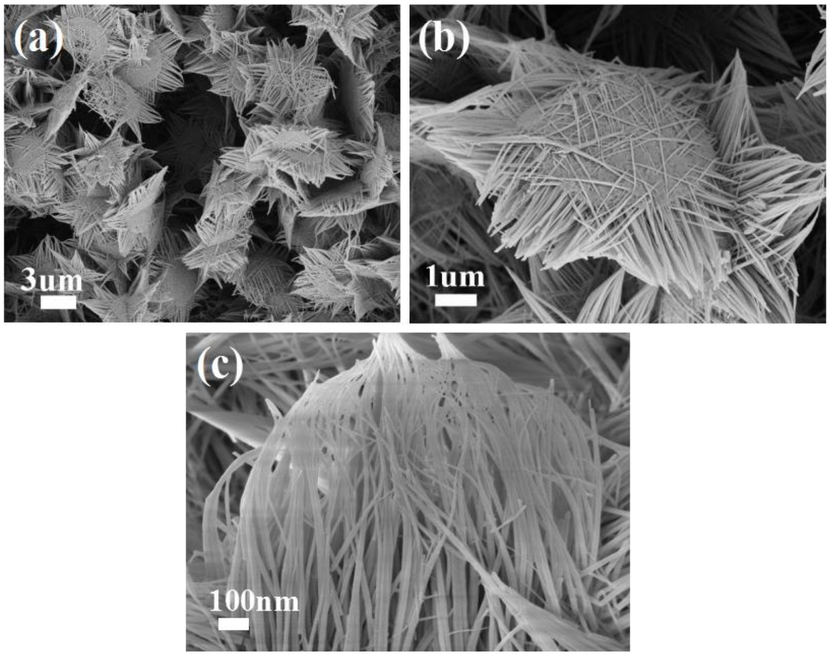

Fig. S4 a-c SEM images of CoMn-LDH/Ni in different magnifications

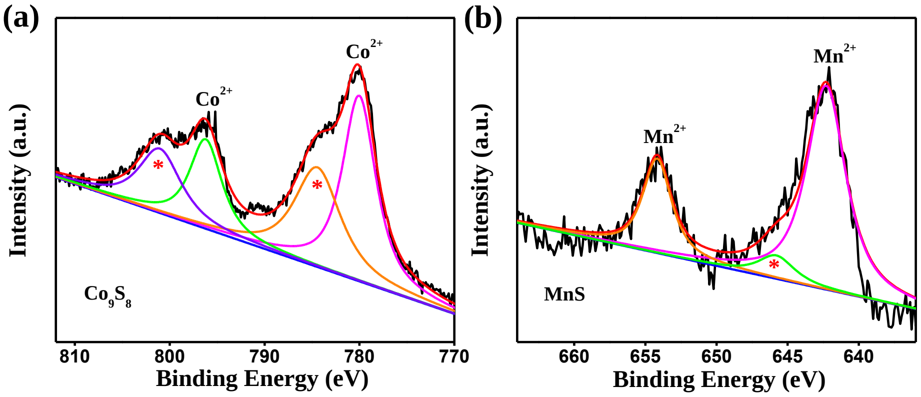

Fig. S5 a Co 2p XPS spectra of Co_9S_8/Ni; **b** Mn 2p XPS spectra of MnS/Ni

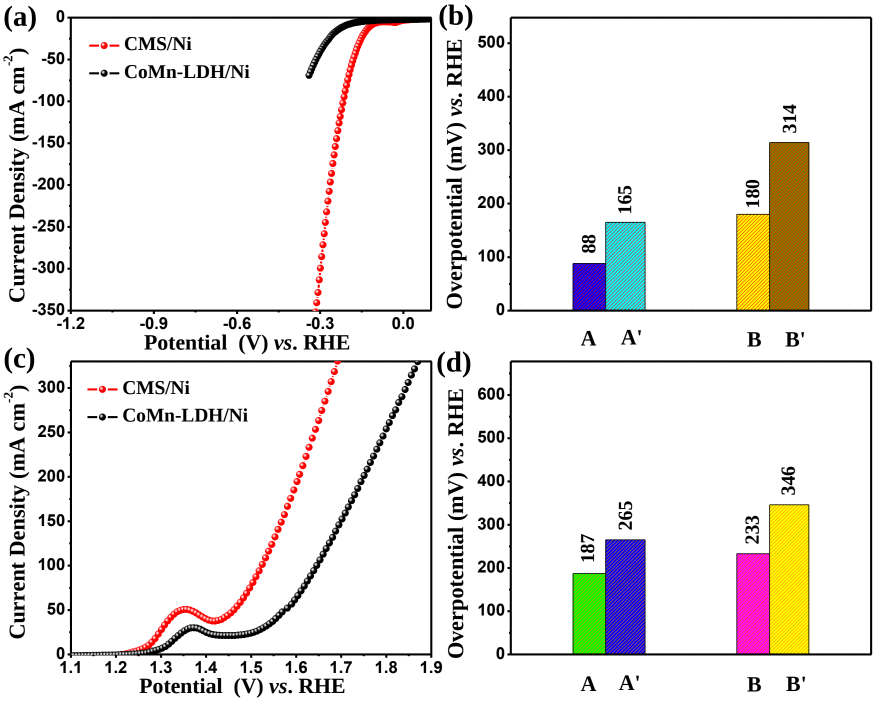

Fig. S6 a LSV curves of CMS/Ni and CoMn-LDH/Ni for HER. **b** A and A' are corresponding to the onset overpotentials of CMS/Ni and CoMn-LDH/Ni for HER, and B and B' are corresponding to the overpotentials of CMS/Ni and CoMn-LDH/Ni to achieve a current density of 50 mA cm^{-2}. **c** LSV curves of CMS/Ni and CoMn-LDH/Ni for OER. **d** A and A' are related to the onset overpotentials of CMS/Ni and CoMn-LDH/Ni for OER, and B and B' are related to the overpotentials of CMS/Ni and CoMn-LDH/Ni to reach a current density of 50 mA cm^{-2}.

As summarized in Fig. S6, the onset overpotentials and overpotentials of CMS/Ni to achieve a current density of 50 mA cm^{-2} for HER and OER are both lower than the CoMn-LDH/Ni, indicating improvement of electrocatalytic activities.

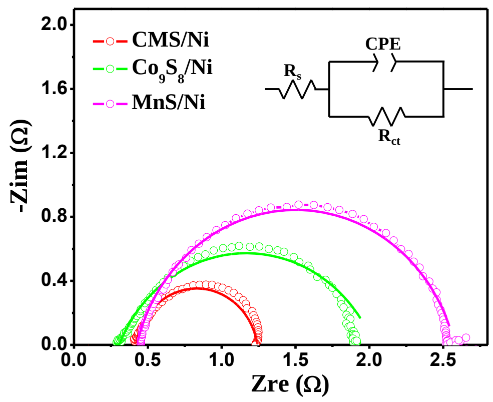

Fig. S7 EIS of CMS/Ni, Co_9S_8/Ni, and MnS/Ni analyzed at a static potential of -0.33 V

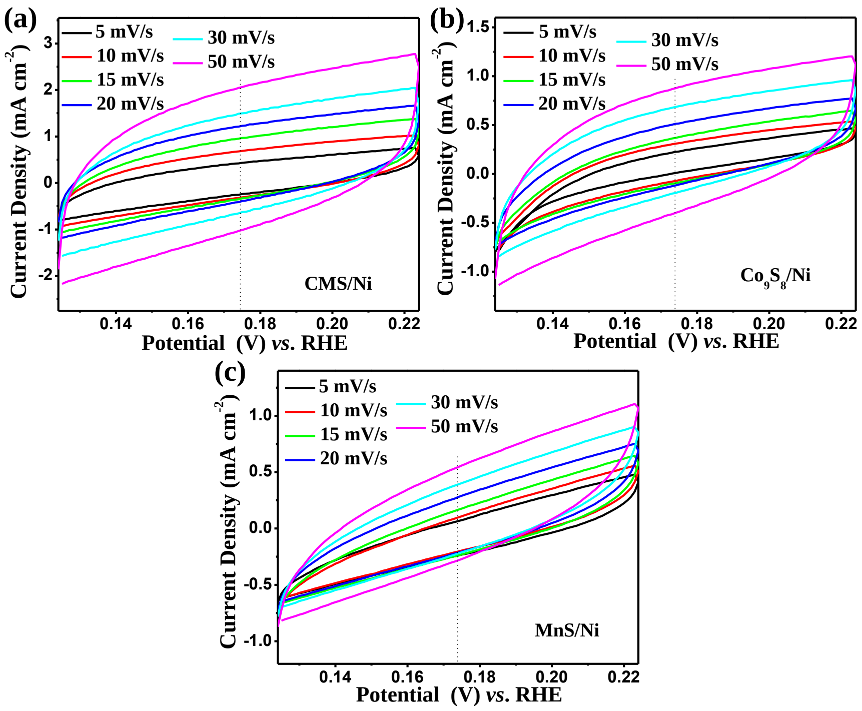

Fig. S8 Cyclic voltammograms of **a** CMS/Ni, **b** Co_9S_8/Ni and **c** MnS/Ni tested at different scan rates of 5, 10, 15, 20, 30, and 50 mV s^{-1}, respectively

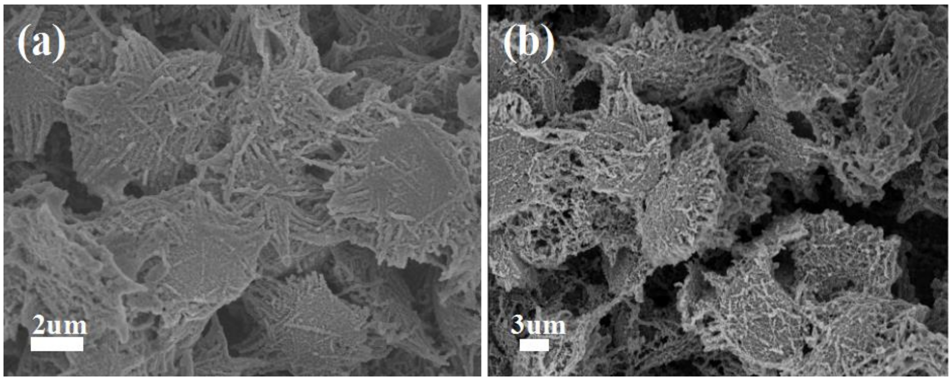

Fig. S9 a SEM images of CMS/Ni after HER and **b** OER stability tests

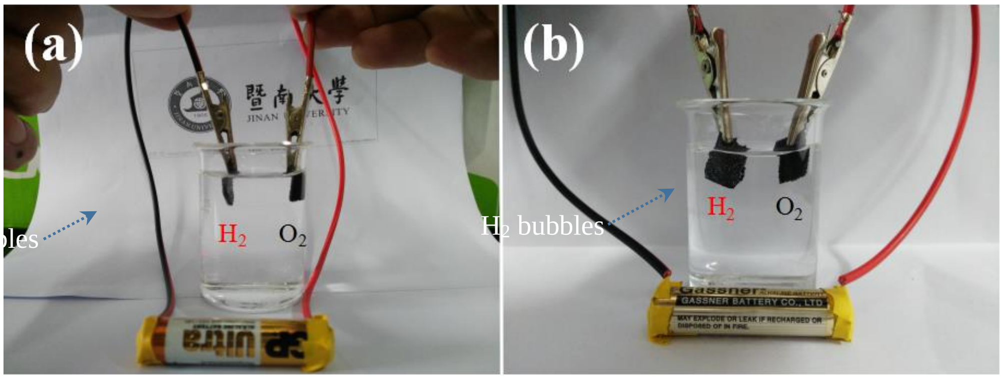

Fig. S10 Photographs of CMS/Ni//CMS/Ni device driven by a 1.5 V dry battery. The white bubbles of H_2 can be obviously observed in cathode, while the O_2 has not enough bubbles simultaneously, attributing to its kinetically sluggish four-electron transfer process

Table S1 Comparison of catalytic activity of CMS/Ni to recently reported bifunctional materials for OER, HER, and overall water splitting

Materials	HER η_j=100 mA cm^{-2} (mV *vs.* RHE)	OER η_j=100 mA cm^{-2} (mV *vs.* RHE)	Two-electrode system E_j=10 mA cm^{-2} (V *vs.* RHE)	Electrolytes (KOH)	Ref.
CMS/Ni	217	292	1.60	1 mol L^{-1}	This work
Zn-Co-S/TM[a]	>330	>340	1.66	1 mol L^{-1}	[2]
PCPTF[b]	>430	>330	/	1 mol L^{-1}	[3]
Co@Co_3O_4-NC[c]	>320	>391	2.00	1 mol L^{-1}	[4]
Ni_3FeN-NPs[d]	>260	>320	/	1 mol L^{-1}	[5]
$NiCo_2S_4$@NiFe LDH/NF[e]	>220	<292	1.60	1 mol L^{-1}	[6]
$SrNb_{0.1}Co_{0.7}Fe_{0.2}O_{3-\delta}$	>300	>350	1.68	1 mol L^{-1}	[7]
CP/CTs/Co-S[f]	>252	>296	~1.74	1 mol L^{-1}	[8]
CoP_3 CPs[g]	>217	>343	/	1 mol L^{-1}	[9]
CoP-MNA[h]	>252	>300	1.62	1 mol L^{-1}	[10]
Co@CoO/NG[i]	>217	>315	1.58	1 mol L^{-1}	[11]
FeCoNi	>220	>325	~1.69	1 mol L^{-1}	[12]
Ni_2P	215	393	1.58	1 mol L^{-1}	[13]
$Ni_{12}P_5$	295	360	1.64	1 mol L^{-1}	[13]

[a] $Zn_{0.76}Co_{0.24}S/CoS_2$ on Ti mesh; [b] porous Co phosphide/phosphate thin film; [c] N-carbon; [d] Nanoparticles, [e] Ni foam; [f] carbon paper/carbon tubes/cobalt-sulfide sheets; [g] concave polyhedrons; [h] mesoporous nanorod arrays; [i] N-doped graphene.

References

[1] G.-F. Chen, T.Y. Ma, Z.-Q. Liu, N. Li, Y.-Z. Su, K. Davey, S.-Z. Qiao, Efficient and stable bifunctional electrocatalysts Ni/Ni_xM_y (M = P, S) for overall water splitting. Adv. Funct. Mater. **26**, 3314-3323 (2016). doi:10.1002/adfm.201505626

[2] Y. Liang, Q. Liu, Y. Luo, X. Sun, Y. He, A.M. Asiri, $Zn_{0.76}Co_{0.24}S/CoS_2$ nanowires array for efficient electrochemical splitting of water. Electrochim. Acta **190**, 360-364 (2016). doi:10.1016/j.electacta.2015.12.153

[3] Y. Yang, H. Fei, G. Ruan, J.M. Tour, Porous cobalt-based thin film as a bifunctional catalyst for hydrogen generation and oxygen generation. Adv. Mater. **27**, 3175-3180 (2015). doi:10.1002/adma.201500894

[4] C. Bai, S. Wei, D. Deng, X. Lin, M. Zheng. Q. Dong, A nitrogen-doped nano carbon dodecahedron with Co@Co_3O_4 implants as a bi-functional electrocatalyst for efficient overall water splitting. J. Mater. Chem. A **5**, 9533-9536 (2017). doi:10.1039/c7ta01708a

[5] X. Jia, Y. Zhao, G. Chen, L. Shang, R. Shi, X. Kang, G.I.N. Waterhouse, L.-Z. Wu, C.-H. Tung, T. Zhang, Ni_3FeN nanoparticles derived from ultrathin NiFe-layered double hydroxide nanosheets: an efficient overall water splitting electrocatalyst. Adv. Energy Mater. **6**, 1502585 (2016). doi:10.1002/aenm.201502585

[6] J. Liu, J. Wang, B. Zhang, Y. Ruan, L. Lv, X. Ji, K. Xu, L. Miao, J. Jiang, Hierarchical $NiCo_2S_4$@NiFe LDH heterostructures supported on nickel foam for enhanced overall-water-splitting activity. ACS Appl. Mater. Interfaces **9**, 15364-15372 (2017). doi:10.1021/acsami.7b00019

[7] Y. Zhu, W. Zhou, Y. Zhong, Y. Bu, X. Chen, Q. Zhong, M. Liu, Z. Shao, A perovskite nanorod as bifunctional electrocatalyst for overall water splitting. Adv. Energy Mater. **7**, 1602122 (2017). doi:10.1002/aenm.201602122

[8] J. Wang, H.X. Zhong, Z.L. Wang, F.L. Meng, X.B. Zhang, Integrated three-dimensional carbon paper/carbon tubes/cobalt-sulfide sheets as an efficient electrode for overall water splitting. ACS Nano **10**, 2342-8 (2016). doi:10.1021/acsnano.5b07126

[9] T. Wu, M. Pi, X. Wang, D. Zhang, S. Chen, Three-dimensional metal-organic framework derived porous CoP_3 concave polyhedrons as superior bifunctional electrocatalysts for the evolution of hydrogen and oxygen. Phys. Chem. Chem. Phys. **19**, 2104-2110 (2017). doi:10.1039/c6cp07294a

[10] Y.-P. Zhu, Y.-P. Liu, T.-Z. Ren, Z.-Y. Yuan, Self-supported cobalt phosphide mesoporous nanorod arrays: a flexible and bifunctional electrode for highly active electrocatalytic water reduction and oxidation. Adv. Funct. Mater. **25**, 7337-7347 (2015). doi:10.1002/adfm.201503666

[11] S. Zhang, X. Yu, F. Yan, C. Li, X. Zhang, Y. Chen, N-doped graphene-supported Co@CoO core-shell nanoparticles as high-performance bifunctional electrocatalysts for overall water splitting. J. Mater. Chem. A **4**, 12046-12053 (2016). doi:10.1039/c6ta04365h

[12] Y. Yang, Z. Lin, S. Gao, J. Su, Z. Lun, G. Xia, J. Chen, R. Zhang, Q. Chen, Tuning electronic structures of nonprecious ternary alloys encapsulated in graphene layers for optimizing overall water splitting activity. ACS Catal. **7**, 469-479 (2017). doi:10.1021/acscatal.6b02573

[13] P.W. Menezes, A. Indra, C. Das, C. Walter, C. Göbel, V. Gutkin, D. Schmeiβer, M. Driess, Uncovering the nature of active species of nickel phosphide catalysts in high-performance electrochemical overall water splitting. ACS Catal. **7**, 103-109 (2016). doi:10.1021/acscatal.6b02666
